# The effect of cooling rate and supplying water condition on the freezing properties of silty clay in seasonal freezing zones

**DOI:** 10.1371/journal.pone.0333101

**Published:** 2025-10-06

**Authors:** Haotian Guo, Haojie Qi, Enliang Wang, Peijin Wang, Tao Song, Chao Sun

**Affiliations:** 1 School of Water Conservancy and Civil Engineering, Northeast Agricultural University, Harbin, China; 2 School of Geometrics and Prospecting Engineering, Jilin Jianzhu University, Changchun, China; 3 Seasonal frozen soil engineering disaster mechanism and detection and prevention technology innovation base, Jilin Jianzhu University, Changchun, China; 4 \Heilongjiang Provincial Key Laboratory of Water Resources and Water Conservancy Engineering in Cold Region, Northeast Agricultural University, Harbin, China; King Saud University / Zagazig University, EGYPT

## Abstract

A unidirectional freezing experiment was performed on silty clay from the Changchun area using a custom freezing apparatus to examine temperature variations, frost deformation, and moisture migration within the soil samples. The study aimed to investigate the influence of cooling rate and water supply conditions on the soil’s frost heave properties. The results indicated that under different cooling rates and water supply conditions, the internal temperature evolution of the specimens could be categorized into four distinct stages: rapid cooling, rebound and stabilization, sustained cooling, and steady state.The final temperature of specimens under open water supply conditions was 2–3°C higher than that under closed conditions. The cryostructure exhibited a reticulated pattern, and its width increased from 3–4 cm to 6–7 cm with increasing cooling rate under the same water supply condition. The cooling rate significantly affected the frost heave ratio: under closed conditions, the ratio decreased, whereas under open water supply conditions, the vertical frost heave displacement increased with higher cooling rates.Moisture migration within the specimen was notably different under the two replenishment conditions. Under open water replenishment, the water content of the soil increased near the negative-temperature top plate and decreased in the surrounding areas.

## Introduction

China is the third-largest country in the world in terms of permafrost, with the seasonal frozen soil region covering about 74% of the country’s land area [1]. As the seasons change, soils in these areas undergo a cycle of freezing in winter and thawing in summer. The occurrence of frost heave, frost cracking, and secondary soil damage during the winter is influenced by fluctuations in external temperature and the water table. These processes can cause significant disruptions to engineering projects in seasonal frozen soil regions, affecting both safety and economic aspects [2]. Several researchers have studied the principles of soil freezing by examining soil-water characteristic curves under various soil types and influencing factors [3–7]. Additionally, many scholars have conducted experiments to investigate the phenomena of soil freezing and moisture migration.

Tianxiao Tang et al [8] conducted indoor horizontal freezing experiments to investigate the effects of temperature gradients and initial moisture content on freezing characteristics. Zhang Ting et al. [9] focused on shallow surface soil in the Nanjing area, performing unidirectional freezing experiments to analyze moisture migration in the soil under various test conditions. Wang Tiexing et al. [10] used a homemade moisture migration apparatus, with and without grids inside the test setup, to examine the moisture migration process during soil freezing. Their findings showed that soil density, moisture content, and time all play significant roles in the moisture migration process. Water content, as a key factor in soil freezing and expansion, was highlighted in several studies. When the temperature gradient at the top and bottom of the soil or test samples differs, the freezing fronts within the soil move at different rates, causing variations in moisture migration and ultimately resulting in different freezing and expansion outcomes [11]. Mo Chen et al. [12] conducted indoor experiments on unsaturated soils, studying the effects of freezing temperature, moisture content, and other factors on moisture migration. Wang YT et al. [13] performed a unidirectional freezing experiment on saturated chalky clay in the Qinghai-Tibet Plateau, providing valuable insights into the dynamic processes of cold-induced tectonic development and freeze-up behavior. Xue Ke et al. [14]reviewed the current progress in moisture migration research in unsaturated soils in cold regions, offering a comprehensive explanation of the driving forces behind moisture migration. Their research indicated that various factors such as soil properties, moisture content, temperature, infill methods, and overlying loads can all influence moisture migration to different extents. Additionally, Hao Xiaoyun et al. [15] conducted moisture migration tests on calcareous, clayey, and sandy soils in Qinghai, Tibet, incorporating pressurized water supply conditions as one of the test parameters. Their results confirmed that pressurized water supply significantly affects soil freezing and expansion.

Scientists have studied the effects of various factors such as overburden loads [16], cold end temperatures [17], recharge pressures, soil quality, freeze-thaw cycles, moisture content, and more on moisture migration and its mechanisms [18–22]. Numerical simulation techniques continue to evolve, with an increasing body of research exploring frost heave modeling in greater depth and sophistication [23]. However, few studies have simulated the effects of real-world environmental factors, such as cooling rate and water supply conditions, on soil deformation and moisture migration during freezing. This study addresses this research gap by conducting unidirectional freezing experiments under simulated natural cooling conditions to examine the effects of cooling rate and water supply on the freezing behavior—including temperature changes, frost heave, and moisture migration—of silty clay from Changchun.

## Soil samples and test equipment, and programmes

### Preparation of soil samples

**Fig 1:**
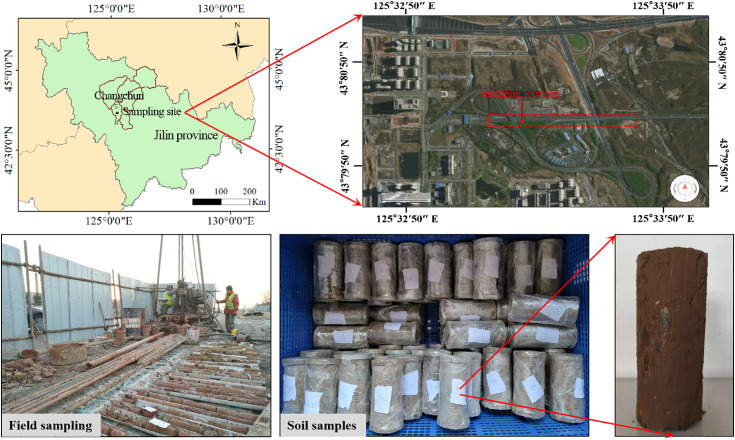
Soil sample preparation process.

The soil samples used in this study were obtained from the construction site of the southern extension of Changchun Rail Transit Line 1. The in-situ soils were then subjected to basic physical tests according to ASTM standards. The basic physical properties of the soil are presented in Table 1. After collection, the native soil was dried, crushed, and sieved through a 2 mm sieve. To prepare the soil samples for testing, they were adjusted to match the moisture content of the in situ soil. The samples were placed in airtight containers for 24 hours to allow the moisture to distribute evenly throughout the soil. Following this resting period, place the prepared soil in the compaction cylinder shown in Fig 1 and compact it in three stages, with 25 compaction strokes each time. The compaction layer thickness is approximately 3.5 cm. After making two samples, place them in an acrylic cylinder with a height of 23 cm. Trim off any excess material according to the cylinder dimensions and ensure the surface is flat. The final test specimen had a height of 23 cm and a diameter of 10 cm. The preparation process is shown in Fig 1.No permits were required for the present work. The field sites involved in this study are non-protected public areas, and the research activities conducted do not involve protected species, ecosystems under special regulations, or any operations that require specific authorization as stipulated by relevant national or local regulations. Therefore, in accordance with the applicable rules, no permits were necessary for accessing the field sites or carrying out the work.

**Table 1:** Basic physical properties of soil samples.

Liquid limit/%	Plastic limit./%	Plasticity Index	Fluidity Index	Average moisture level/%	Average Density/ (${g/cm}^{-3}$)	dry density/ (${g/cm}^{-3}$)
32.9	21.85	12.82	0.20	26.54	1.93	1.65

### Test equipment and programmes

**Fig 2:**
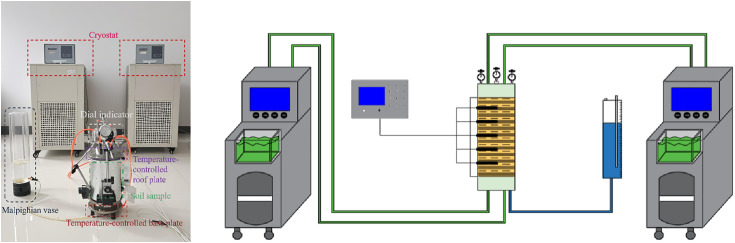
Test equipment.

The moisture migration test apparatus is depicted in Fig 2.The unidirectional freezing test device mainly consists of a JK4000 multi-channel temperature acquisition instrument, an insulation box, low-temperature thermostatic baths, and a sample cylinder. The sample cylinder contains temperature-controlled top and bottom plates, a dial gauge, a temperature sensor, and an acrylic sleeve. The low-temperature thermostatic baths are products of Xi’an Yaxing Civil Engineering Instrument Factory, with models DC-2020 and DC-4020. Both are equipped with a circulation system, which can lead out antifreeze to establish a second constant temperature field. They have a programming function, allowing the setting of temperature and the time to reach the set temperature, and can store up to 30 temperature segments, supporting unmanned automatic operation. The temperature control range of DC-2020 is −20 ~ 100°C, and that of DC-4020 is −40 ~ 100°C, with both achieving a control accuracy of 0.01°C. The circulating fluid uses antifreeze with a freezing point ≤ −45°C, meeting the temperature requirements of all tests. The temperature-controlled top and bottom plates are stainless steel hollow structures (facilitating the flow of circulating fluid): the water inlet and outlet of the top plate are connected to DC-4020 to form a closed loop, enabling circulating cooling of the antifreeze; the bottom plate is connected to DC-2020, with the same working principle. Three percentage gauges, accurate to 0.01 mm, were positioned at the top of the test rig to monitor the vertical freezing displacement of the specimen at the end of the test. Temperature sensors were placed along the longitudinal axis of the soil samples, with the top sensor positioned 3 cm from the top plate and the remaining sensors spaced 4 cm apart. The specimen sleeve was surrounded by an insulating material to ensure unidirectional freezing. To simulate the process of atmospheric temperature transfer from the top to the deeper layers of the soil, we conducted tests under two conditions: an open water replenishment condition and a closed, no-supplied water condition. In the rehydration test, the sample was rehydrated without pressure using a Martesian bottle, ensuring the water reached the bottom of the sample. During the test, the temperature of the temperature-controlled bottom plate was set to 3°C, while the temperature of the top plate was set to −15°C.Our study area is Changchun, a typical seasonal frozen soil region. The experimental temperature settings refer to the actual engineering environmental characteristics of the local area:Top plate at −15°C: Simulates the extreme low temperatures in Changchun during winter (often reaching −15°C to −18°C), reflecting the low-temperature environment exposed to surface soil;

Bottom plate at 3°C: Simulates the temperature of deep soil (below the freezing depth), where the deep soil in this region mostly remains unfrozen at 2–5°C in winter, forming a realistic freezing gradient.

The thermostatic baths were programmed following sample installation. Prior to initiating unidirectional freezing, the temperatures of both the top and bottom plates were set to +10°C and maintained for 60 minutes to establish a uniform initial temperature field.Afterward, the cooling time was controlled (30 min, 60 min, 90 min) to allow the circulating liquid in the cryostat to reach the specified negative temperature. This approach enabled us to effectively study the effects of different cooling rates on soil freezing and expansion. The experimental program is summarized in Table 2.Changchun is located in the middle temperate zone with a typical seasonal frozen soil environment. According to long-term meteorological data, the minimum winter temperature in Changchun can reach −20°C, and the average extreme low temperature during the freezing period (December to February) is generally between −15°C and −18°C (consistent with the climatic characteristics of seasonal frozen soil regions described in the introduction: “soils in these areas undergo a cycle of freezing in winter and thawing in summer”). The top plate in the experiment simulates the atmospheric temperature boundary, where the soil is directly exposed to cold air. Setting the top plate temperature to −15°C is intended to mimic the actual low-temperature environment during the peak freezing period in Changchun, ensuring that the experimental conditions reflect the real cooling intensity experienced by surface soils in engineering projects (e.g., rail transit subgrades, as the soil samples were collected from Changchun Rail Transit Line 1 extension).

**Table 2:** Test Schedule.

Supplying water conditions	Soil sample number	base plate temperature/°C	top plate temperature/°C	cooling time/min	Initial moisture content/%
Closed systems	T1	3	−15	30	26.5
	T2	3	−15	60	26.5
	T3	3	−15	90	26.5
Open systems	D1	3	−15	30	26.5
	D2	3	−15	60	26.5
	D3	3	−15	90	26.5

The bottom plate temperature of 3°C is designed to simulate the temperature of deep soil layers in seasonal frozen zones. In practical engineering environments, deep soil (below the maximum freezing depth) is less affected by atmospheric temperature fluctuations and maintains a relatively stable temperature above the freezing point during winter. For Changchun, the maximum seasonal freezing depth is approximately 1.5–2.0 meters, and soil below this depth typically remains unfrozen with temperatures ranging from 2°C to 5°C (consistent with the “unidirectional freezing” process described in the study, where freezing propagates from the surface to depth). Setting the bottom plate to 3°C creates a realistic vertical temperature gradient (from −15°C at the top to 3°C at the bottom), which drives the unidirectional freezing and moisture migration processes, accurately replicating the natural freezing behavior of soil in engineering sites.

## Test results and analyses

### Changes in the internal temperature of the soil

**Fig 3:**
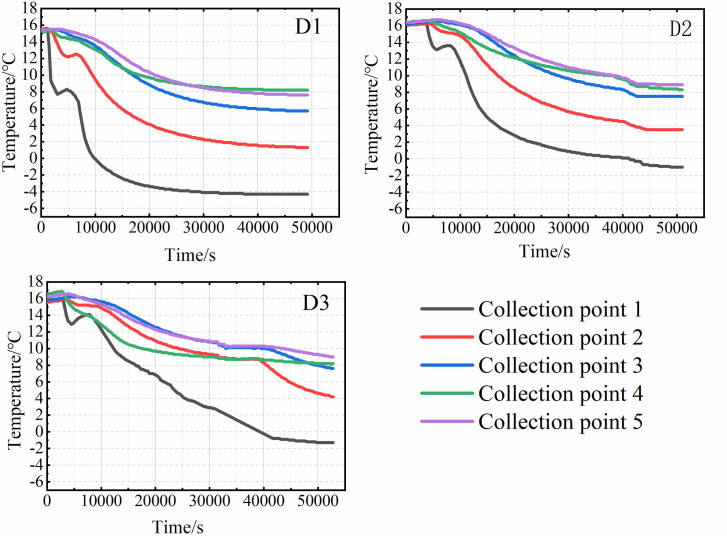
Soil temperature variation curve of the closed system.

**Fig 4:**
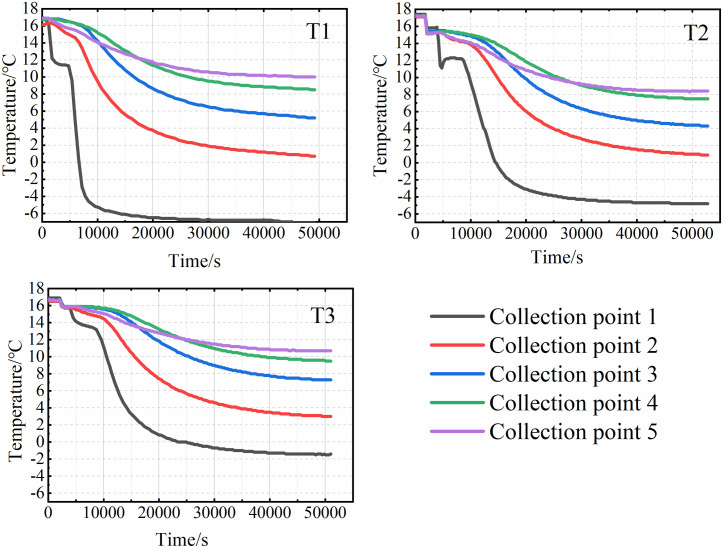
Soil temperature variation curve of the water supplement system.

Based on the temperature monitoring data, variation curves for each sampling point were plotted. The results for the three specimens under closed conditions are shown in Fig 3, and those for the three specimens under open water supply conditions are presented in Fig 4. The results indicate that the internal soil temperature evolution during cooling can be divided into four distinct stages. Stage I is characterized by a rapid temperature decrease, lasting approximately 30 minutes. Owing to its proximity to the top plate, the temperature at Sensor 1 (T1) decreased most rapidly, by approximately 4–5°C. This phase was brief. Stage II represents a temperature rebound or equilibration phase, lasting approximately 60 minutes. During this stage, the temperature increased by approximately 1°C, attributed to the latent heat released by moisture migration, although the magnitude of increase was generally ≤0.3°C at most points. Consequently, the soil temperature became slightly higher than that at the end of Stage I or remained relatively constant. Stage III involved sustained cooling, lasting approximately 100 minutes. The cooling rate during this stage decreased to approximately 0.3°C/min. The most pronounced temperature drop occurred at Sensor 1 (T1), where the temperature transitioned to subzero values. Compared to Stage I, the rate of temperature change was slower, resulting in a gentler slope of the temperature-time curve. Stage IV, the final stage, was a steady-state phase where temperatures gradually stabilized, and the rate of change decreased to below 0.0016°C/min.

A comparison of the experimental results from six groups of test specimens reveals that, under open water replenishment conditions, the final temperature inside the test specimens is 2–3°C higher than in the closed system. Furthermore, the temperature evolution patterns differed with sensor depth within a given specimen.

**Fig 5:**
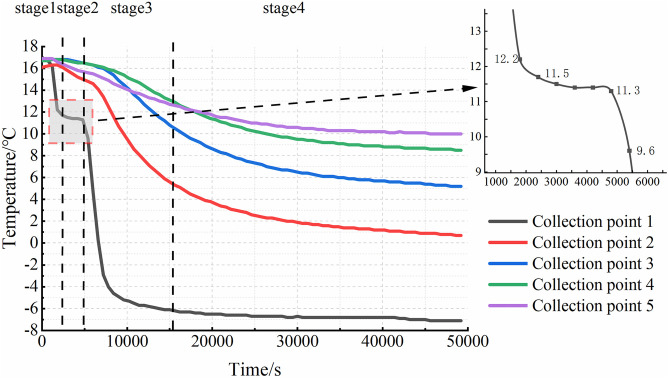
Temperature curve of sample T1.

**Fig 6:**
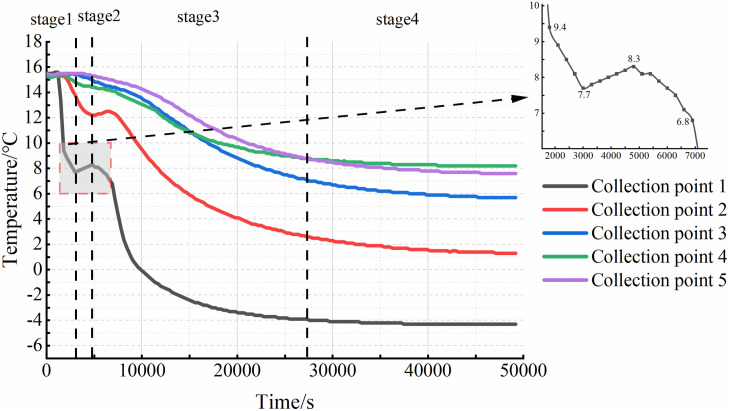
Temperature curve of sample D1.

In the first stage, the temperature at each collection point gradually decreases as the temperature of the top plate drops. The closer a collection point is to the roof, the faster the temperature change. Additionally, the higher the cooling rate, the shorter the duration of this phase. The second stage is primarily influenced by the moisture content within the soil and the surrounding soil layers. As the temperature decreases, moisture from deeper soil layers moves upward due to the combined effects of temperature and soil water potential, transferring heat from the deeper soil to the area near Collection Point 1. This results in a slight increase in temperature. Under closed conditions, the temperature profile during this stage remains relatively flat, as shown in Fig 5. However, under water supply conditions, the internal soil temperature begins to rise, as shown in Fig 6. This temperature rally is mainly due to the presence of the water supply device, which triggers intense water migration within the soil. The heat carried by the water influences the internal soil temperature. As illustrated in Figs 3 and 4, temperatures at other collection sites during the second stage were slightly higher under the water supply condition compared to the closed condition. This suggests that water supply not only affects soil moisture content but also plays a role in heat transfer within the soil. In the third stage, the moisture in the soil gradually freezes, and the temperature continues to drop. As freezing progresses, the rate of temperature decrease slows, and the temperature curve becomes less steep. Once the soil temperature reaches a certain negative value, the temperature change gradually levels off, marking the beginning of the fourth stage. The slower the cooling rate, the longer it takes for the sample to reach this stage. When comparing the results from the closed environment to those under water supply conditions, with the same cooling rate, it is evident that the temperature profiles in the first three stages are relatively flat under the water supply conditions. As a result, the fourth stage accounts for a smaller proportion of the total time in the test.

The test data revealed that different cooling rates led to varying final temperatures at different points in the soil after 12 hours of freezing. When the cooling rate was higher, the energy loss during heat transfer was smaller, allowing the sample to enter the fourth stage more quickly and ultimately reach a lower temperature. Conversely, with a slower cooling rate, the moisture in the soil had more time to freeze, and the phase change of the water to ice released heat. This delayed the transition into the fourth stage, resulting in a higher final temperature at the end of the test.

### Soil freezing characteristics

**Fig 7:**
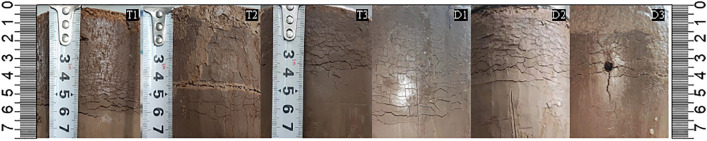
The apparent cold structure of soil.

The tested soil was an unsaturated silty clay from Changchun, exhibiting a dual-porosity structure with both interconnected and isolated pores. The cryostructure within the fully frozen zone exhibited a reticulated (net-like) pattern across all cooling rates. The soil specimens used in the test have a height of 23 cm and a diameter of 10 cm, with unidirectional freezing occurring from the top plate (set at −15°C) to the bottom plate (set at 3°C). The apparent cold structure in Fig 7 reflects the frozen zone of the specimen after 12 hours of freezing, and its distribution range is within the vertical section of the 23 cm high specimen. The cold structure is mainly distributed in the area affected by the freezing front, which advances from the top plate downward. For specific positions relative to the top plate, they roughly correspond to the range where the freezing front propagates.As shown in Fig 7, samples T1–T3 display the apparent cryostructures after 12 hours of freezing at different cooling rates under closed conditions, while D1–D3 correspond to those under open (water-supply) conditions. Fig 7 shows that under open water supply conditions, the width of the cryostructure increased from 4.2 ± 0.2 cm to 5.1 ± 0.3 cm, representing a 21% increase. In contrast, under closed conditions, the width increased from 3.8 ± 0.2 cm to 6.6 ± 0.3 cm (a 74% increase) as the cooling time increased from 30 to 90 minutes. This indicates that the cryostructure development (e.g., ice lens spacing and width) was influenced by both the water supply condition and the cooling rate, despite an identical total freezing duration. Furthermore, the maximum freezing depth was comparable across all specimens under closed conditions. However, a significant difference in maximum freezing depth was observed among the specimens under water-supply conditions. A higher cooling rate resulted in a greater freezing depth after 12 hours. As shown in Fig 7, the difference in the mesh structure morphology was more pronounced for specimens cooled over 60 and 90 minutes than for those cooled over 30 minutes.

The observed phenomena are intrinsically linked to the distribution and migration of moisture within the soil. In unsaturated soils, soil water potential gradients drive moisture migration toward the freezing front (cold end), while also enabling in situ freezing and frost heave. A higher cooling rate promotes faster propagation of the cold front through the soil, leading to more rapid advancement of the freezing front. As freezing progresses, the freezing rate gradually decelerates, and the soil temperature approaches a steady-state equilibrium. During this phase, moisture migration diminishes, promoting more in situ freezing and consequently resulting in less overall frost heave. Conversely, a slower cooling rate facilitates a more gradual thermal penetration and a slower migration of the freezing front. This provides sufficient time for moisture to migrate toward the freezing zone, driven by soil water potential gradients and the prevailing thermal gradient. As water replenishes the frozen zone from below, a significant quantity freezes, and the associated release of latent heat during phase change prolongs the time required to attain thermal equilibrium in the soil.

### Vertical frost heave law of soil

In the unidirectional freezing experiment, when the soil freezes at a constant temperature, there are varying degrees of vertical freezing displacements, which in turn lead to changes in the height of the sample. To quantitatively characterize this frost heave deformation of the soil, the frost heave ratio parameter is calculated:


$$\eta =\frac{\Delta h}{H_{f}}\times \text{1}\text{0}\text{0}\%$$ (1)

η——Frost heave rate(%)

Δh——Amount of frost heave (mm)

$H_{f}$——Deep frozen (Excluding amount of frost heave) (mm)

(The frost heave amount is obtained by the reading difference of the dial indicator before and after the experiment, and the freezing depth is obtained by subtracting the frost heave amount from the distance from the freezing front to the roof observed after the experiment.)

**Fig 8:**
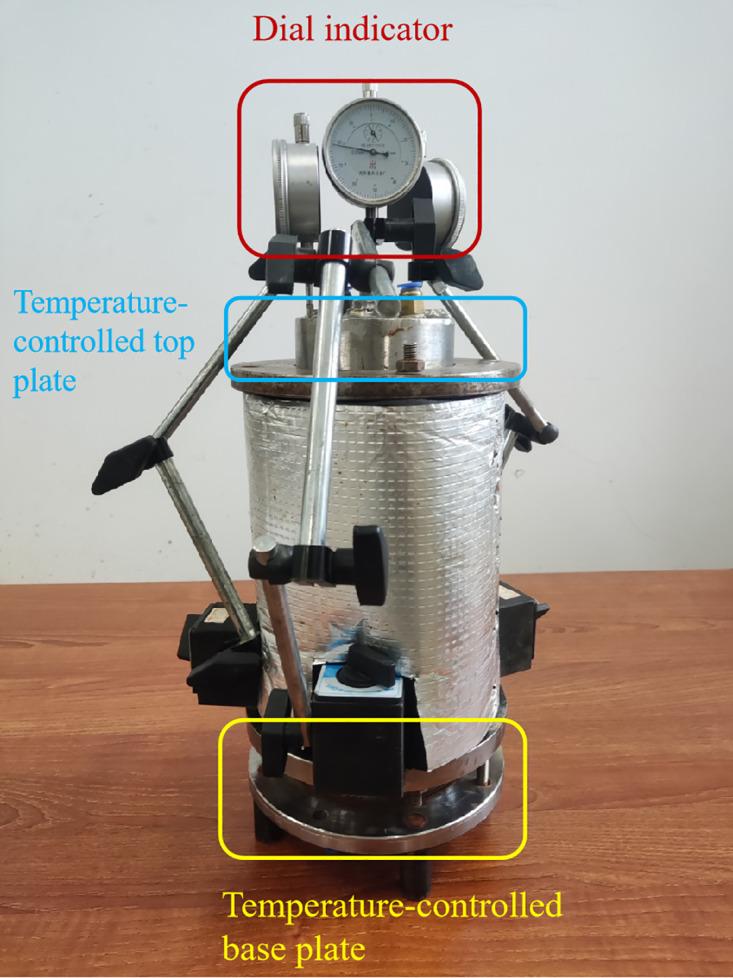
Local schematic diagram of freezing device.

**Fig 9:**
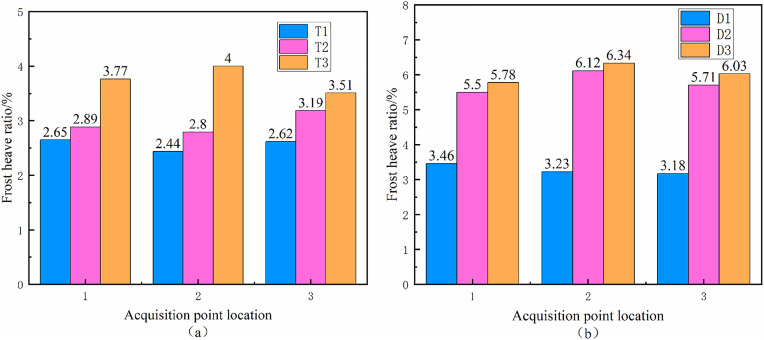
Frost heave rate of different collection points of the sample.

**Fig 10:**
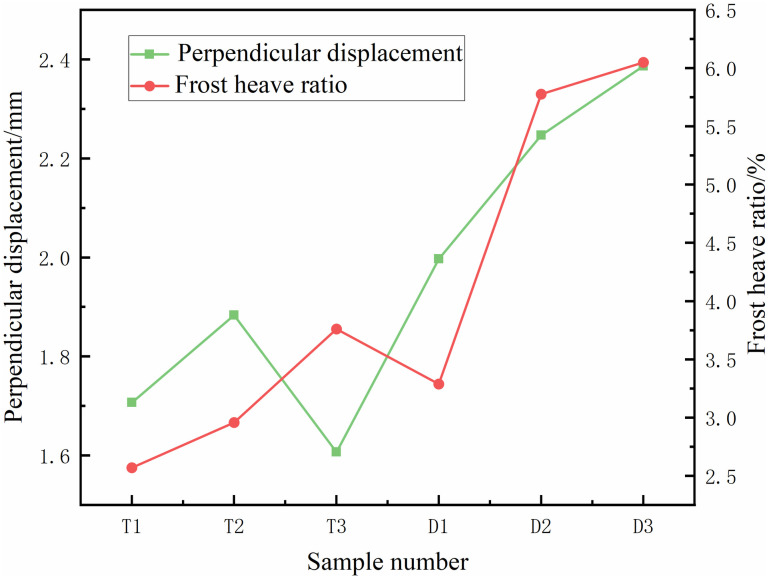
Freezing of soil samples.

**Fig 11:**
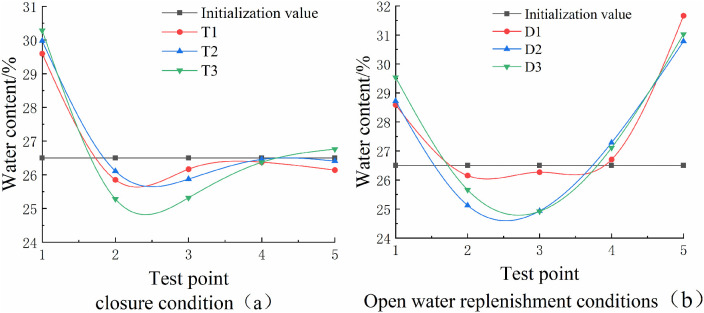
Moisture content change curve.

The tests were conducted using a unidirectional freezing experiment, with the test setup shown in Fig 8. The height of the soil sample is 23 cm. To prevent heat loss during the temperature transfer project, the soil sample is closely fitted to the top/bottom plates.The vertical freezing expansion of the soil was measured using a percentage gauge located at the top of the setup. The average vertical frost heave and the frost heave rate at the three measurement points were compared, as shown in Figs 9–11. Figs 9(a) and 9(b) illustrate the freezing expansion rates of each specimen under closed and open water supply conditions, respectively. From these figures, it is evident that the rate of freezing expansion is similar across different collection points within the same sample.

Under open water supply conditions, the average frost heave displacement was 1.2 ± 0.1 mm, with a frost heave ratio of 0.8% ± 0.05% at a cooling time of 30 minutes. When the cooling time was increased to 90 minutes, the frost heave displacement increased to 1.8 ± 0.15 mm, and the frost heave ratio rose to 1.2% ± 0.08%. The low-temperature transfer process is influenced by the release of heat during the water-ice phase transition, and moisture freezes into ice at varying rates as the temperature decreases. As the soil freezes, the growth of ice crystals compresses the lower pore spaces, increasing the pore water pressure and promoting moisture migration into the freezing zone under pressure. When the cooling rate is low, the freezing front advances more slowly, providing more time for the water in the soil to freeze into ice. This reduces the amount of unfrozen water in the frozen zone, ultimately leading to an increase in the frost heave ratio of the soil.

These trends can be interpreted using the theory of frost heave mechanics. Frost heave is driven by ice crystal growth, which depends on the amount of water available in the freezing zone. Under open conditions, continuous water supply provides more liquid water to form ice, leading to larger displacements than in closed conditions (where moisture is limited). For the same water supply condition, slower cooling rates (90 minutes) allow more time for moisture to migrate to the freezing front (driven by soil water potential gradients), increasing ice volume and thus frost heave ratio—consistent with the concept that moisture migration time is a key factor in ice segregation.

The average vertical freeze-up displacements of the six groups of specimens are presented in Fig 10. As shown, the difference in average vertical freezing displacements between samples with different water supply conditions at the same cooling rate is minimal. However, for the same water supply conditions, the vertical freeze-up displacement of the soil increases as the cooling rate decreases. The difference in vertical freeze-up displacement between the specimens at various cooling rates was approximately 0.2 mm, indicating that the cooling rate has a significant impact on the freezing and expansion behavior of the soil. The frost heave ratio of the soil after 720 minutes of freezing for each sample is shown in Fig 10. Under identical water supply conditions, the frost heave ratio of the sample increases progressively as the cooling rate decreases. This is because, at lower cooling rates, the unfrozen water content in the frozen zone of the sample is reduced, leading to a shallower maximum freezing depth due to the release of heat during the phase change from water to ice. The maximum frost depth of the sample under the supplying water condition is less than that in the closed condition, owing to the influence of the supplying water temperature. These results suggest a negative correlation between the soil frost heave ratio and the cooling rate: higher cooling rates correspond to lower frost heave ratios.

To quantify the relationship between cooling rate and frost heave ratio, the key test data were first extracted and organized (Table 3). The cooling rate was calculated based on the temperature difference and cooling time. The frost heave ratio was obtained from the average value of three dial indicator measurement points (Fig 9) after 720 minutes of freezing, with errors controlled within ±0.05%.

**Table 3:** Statistical table of cooling rate and frost heave ratio under different water supply conditions.

Supplyingwater conditions	Cooling time (min)	Cooling rate (°C/min)	Frost heave ratio (%)
Closed systems	30	0.833	0.52
	60	0.417	0.71
	90	0.278	0.93
Open systems	30	0.833	0.81
	60	0.417	1.02
	90	0.278	1.19

The least square method is used to linearly fit the cooling rate and frost heave ratio under different water replenishment conditions. The fitting results are as follows:

Closed water supply conditions:η=−0.528v+1.031

Open water supply conditions:η=−0.648v+1.347

The fitting coefficients *R*^*2*^ under different water supplement conditions were 0.986 and 0.972, respectively.

The R2values of both fitting models are greater than 0.97, indicating a strong linear correlation between cooling rate and frost heave ratio. The negative coefficients of v (−0.582 for closed conditions, −0.648 for open conditions) quantitatively confirm that the frost heave ratio decreases with increasing cooling rate; meanwhile, the higher intercept of the open condition fitting line (1.347 vs. 1.031) reflects that the open water supply increases the base frost heave ratio, which is consistent with the earlier qualitative observation of “higher frost heave ratio under open conditions”.

### The change of internal moisture content of soil

The influence of soil freezing and expansion is affected by various factors, including not only the external environment but also changes in the moisture state within the soil and the mode of moisture migration. These factors have been a focal point for many researchers over time [23]. After the unidirectional freezing experiment, the sample was divided into axial quintiles, and moisture content was measured in each section to assess the state of moisture and the effect of moisture migration on freezing and expansion. The test results are presented in Figs 11(a) and 11(b). Fig 11(a) shows the results under closed conditions, while Fig 11(b) presents the results under water-supply conditions. The changes in water content within different parts of the soil under the same water-supply conditions exhibit similar trends. The test sections were numbered from top to bottom. Test point 1, located at the uppermost part of the sample, showed an increase in moisture content under both confined and water-supply conditions. Conversely, the data measured at test point 2 showed a decrease in moisture content, indicating moisture migration within the soil closer to the temperature-controlled top plate. The extent of moisture migration was relatively small. Under closed conditions, the average moisture content of all sections (26.4 ± 0.3%) is nearly identical to the initial moisture content (26.54%), with a maximum variation of ±0.5% at any test point. This indicates minimal moisture migration, as the closed system limits water supply, and the fixed moisture mostly undergoes in-situ freezing. Under open conditions, moisture migration is significant: test point 1 (topmost) shows a moisture content increase of 1.2–1.8% compared to the initial value, while test points 4 and 5 (bottom layers) increase by 0.5–3.5% (reaching 27% and 30%, respectively). This upward moisture movement is driven by the combined effect of matrix potential (due to capillary action in soil pores) and temperature potential (water moves toward colder regions).

The higher the cooling rate under the same water supply conditions, the smaller the increase in water content measured at test point 1. This occurs because, at a high cooling rate, the freezing front moves rapidly through the soil, limiting the time for the soil temperature to approach equilibrium. As a result, more in situ freezing happens in the soil. In contrast, when the cooling rate is lower, the moisture in the soil beneath the freezing front moves toward the cold source, and the unfrozen water content in the frozen region above the freezing front is reduced. The heat released during the water-to-ice phase transition also slows the movement of the freezing front. Consequently, the moisture in the lower soil layers has more time to migrate toward the freezing front, leading to a larger increase in water content at test point 1 as the cooling rate decreases. At test points 4 and 5, the increase in water content under the supplied water condition indicates moisture migration within the soil. In this case, the migration is relatively unaffected by the temperature environment, with the main driving force being the connectivity of the soil’s internal pores.

## Conclusion

This study investigated the unidirectional freezing behavior of silty clay, typical of seasonal frozen regions, focusing on the effects of cooling rate and water supply conditions. The main findings are as follows:

(1) The internal temperature evolution under different cooling rates and water supply conditions followed a similar four-stage pattern: rapid cooling, rebound and stabilization, sustained cooling, and steady state. The final soil temperature under open water supply conditions was 2–3°C higher than at equivalent depths under closed conditions. Under identical water supply conditions, a higher cooling rate accelerated heat transfer, shortening the combined duration of the first three stages and resulting in a lower final specimen temperature.
(2) The apparent cryostructure exhibited a reticulated pattern in all tests. The width of the reticulated structure was significantly influenced by both the cooling rate and the water supply condition. For a given cooling rate, the cryostructure width was greater under closed conditions than under open water supply conditions. For a given water supply condition, a higher cooling rate produced a wider cryostructure.
(3) Both the cooling rate and water supply condition significantly influenced the freezing depth and the frost heave ratio. Under open conditions, the final freezing depth was lower than under closed conditions, attributed to the thermal effect of the supplied water. Closed-system specimens exhibited a greater freezing depth but a lower frost heave ratio than open-system specimens. For a given water supply condition, a higher cooling rate resulted in a greater freezing depth and a lower frost heave ratio.
(4) The cooling rate governs the frost heave ratio by controlling the coupling between the thermal regime and moisture migration:A higher cooling rate accelerates freezing front advancement, curtailing the time available for moisture migration. This limits ice lens growth (promoting in situ freezing) and reduces the frost heave ratio. A lower cooling rate slows the freezing front progression, providing sufficient time for moisture to migrate to the freezing front, driven by matric and thermal potential gradients. Abundant migrated water facilitates extensive ice lens formation, thereby increasing the frost heave ratio. In closed systems, the limited moisture availability amplifies this effect, resulting in lower overall frost heave ratios. In open systems, continuous water supply provides additional moisture for heave, resulting in higher absolute frost heave ratios than in closed systems, though the same inverse relationship with cooling rate is maintained.

The experimental setup effectively simulated key field conditions, providing valuable insights into the freezing and moisture migration behavior of silty clay under controlled laboratory conditions. These findings offer a reliable experimental basis for informing frost heave mitigation strategies in actual engineering design. This study focused on a constant water supply and a single freeze cycle. Future work should incorporate dynamic water pressure conditions and multiple freeze-thaw cycles to validate the findings further and enhance their applicability to real-world engineering scenarios.

By simulating the cooling environment of the actual environment, this paper analyzes the interaction between the cooling rate and the water replenishment conditions, and the influence on the freezing characteristics of silty clay widely distributed in the typical seasonal frozen area-Changchun area. Whether the conclusions are applicable to other types of soil will be clarified in the follow-up study.

## Supporting information

S1 Data (Rar)

## References

[1] Zhang LH, Ma W, SHi YJ, Huang YT, Han DW, Yang CS. The modes and its implications of water accumulation near the freezing front during soil freezing with considering ice segregation. Journal of Glaciology and Geocryology. 2023;45(01):31–41.

[2] Wang YT, Wang DY, Guo Y, Lei LL, Gu TX. Experimental study of the development characteristic of frost heaving ratio of the saturated Tibetan silt under one-dimensional freezing. Journal of Glaciology and Geocryology. 2016;38(02):409–15.

[3] Teng J, Kou J, Yan X, Zhang S, Sheng D. Parameterization of soil freezing characteristic curve for unsaturated soils. Cold Regions Science and Technology. 2020;170:102928. doi: 10.1016/j.coldregions.2019.102928

[4] Vu QH, Pereira J-M, Tang AM. Effect of fines content on soil freezing characteristic curve of sandy soils. Acta Geotech. 2022;17(11):4921–33. doi: 10.1007/s11440-022-01672-9

[5] Ma T, Wei C, Xia X, Zhou J, Chen P. Soil Freezing and Soil Water Retention Characteristics: Connection and Solute Effects. J Perform Constr Facil. 2017;31(1). doi: 10.1061/(asce)cf.1943-5509.0000851

[6] Li X, Zheng S-F, Wang M, Liu A-Q. The prediction of the soil freezing characteristic curve using the soil water characteristic curve. Cold Regions Science and Technology. 2023;212:103880. doi: 10.1016/j.coldregions.2023.103880

[7] Zhou B, Brouchkov AV, Eremina LI, Xu C, Hu J. Numerical Simulation of Water Migration during Soil Freezing and Its Resulting Characterization. Applied Sciences. 2024;14(18):8210. doi: 10.3390/app14188210

[8] Tang T, Shen Y, Liu X, Zhang Z, Xu J, Zhang Z. The effect of horizontal freezing on the characteristics of water migration and matric suction in unsaturated silt. Engineering Geology. 2021;288:106166. doi: 10.1016/j.enggeo.2021.106166

[9] Zhang T, Yang P. Effects of unilateralist freezing on the moisture migration of soil. Journal of Nanjing Forestry University (Natural Sciences Edition). 2013;37(01):117–21.

[10] Wang TX, Wang JJ, Zhang LD. Study on classification of sulfate saling soil the highway engineering. Journal of Xi’an University of Architecture & Technology (Natural Science Edition). 2012;44(01):7–13. doi: 10.15986/j.1006-7930.2012.01.002

[11] Chen AJ, Zhang JS, Chen JH, Ming F. Experimental study on moisture migration in remolded clay during unilateral freezing. Journal of Nanjing Forestry University (Natural Sciences Edition). 2016;115–20.

[12] Chen M, Mei J, Shen K, Gao Y. Response of Sandy Soil–Water Migration to Different Conditions under Unidirectional Freezing. Sustainability. 2024;16(9):3597. doi: 10.3390/su16093597

[13] Wang YT, Wang DY, Ma W, Mu YH, Guan H, Gu TX. Experimental study of development of cryostructure and frost heave of the Qinghai-Tibet silty clay under one-dimensional freezing. Rock and Soil Mechanics. 2016;37(05):1333–42. doi: 10.16285/j.rsm.2016.05.015

[14] Xue K, Zheng T, Zhang ML, Liu JP, Yang F. Research progress of mixed water transport in unsaturated soils of cold region. Journal of Hohai University (Natural Sciences). 2022;50(03):39–46.

[15] Hao XY, Feng WJ, Ma W, Wen Z, Zhang LH, Wang YR. Experimental study on the effect of boundary hydraulic pressure on frost heaving of soil. Journal of Glaciology and Geocryology. 2022;44(02):708–16.

[16] Bai R, Lai Y, Zhang M, Jiang H. Investigating the thermo-hydro-mechanical behavior of loess subjected to freeze–thaw cycles. Acta Geotech. 2024;19(9):6305–18. doi: 10.1007/s11440-024-02306-y

[17] Bai R, Lai Y, Pei W, Zhang M. Study on the frost heave behavior of the freezing unsaturated silty clay. Cold Regions Science and Technology. 2022;197:103525. doi: 10.1016/j.coldregions.2022.103525

[18] Wen Z, Deng YS, Feng WJ, Zhirkov A, Zhang LH, Gao Q. Study on the mechanism of moisture migration in freezing soils: review and prospect. Journal of Glaciology and Geocryology. 2023;45(02):588–98.

[19] Zhang YZ, Liu WL, Wang HY, Zhang LH, Chen SJ, Zhu XD. Macro-micro experimental investigation of the initial water content influence on water migration of coarse-grained soil subjected to freezing and thawing. Journal of Glaciology and Geocryology. 2022;44(02):591–601.

[20] Yang P, Wang Y, Z D, Diao PC. Influence of Load on Frost Heaving Characteristics of Remolded Silty Clay. Journal of Zhengzhou University (Engineering Science). 2022;43(06):83–9. doi: 10.13705/j.issn.1671-6833.2022.06.001

[21] Chen H, Li X, Xiong H, Chen X, Su D. A model of segregation frost heave for saturated soil freezing under overburden pressure. Cold Regions Science and Technology. 2023;214:103935. doi: 10.1016/j.coldregions.2023.103935

[22] Zhang H, Wang TX, Luo Y. Experimental study on moisture migration of unsaturated loess under freezing effect. Journal of Engineering Geology. 2015;23(01):72–7. doi: 10.13544/j.cnki.jeg.2015.01.011

[23] Fu Z, Wu Q, Zhang W, He H, Wang L. Water Migration and Segregated Ice Formation in Frozen Ground: Current Advances and Future Perspectives. Front Earth Sci. 2022;10. doi: 10.3389/feart.2022.826961

